# Cholera risk factors, Papua New Guinea, 2010

**DOI:** 10.1186/1471-2334-12-287

**Published:** 2012-11-05

**Authors:** Alexander Rosewell, Benita Addy, Lucas Komnapi, Freda Makanda, Berry Ropa, Enoch Posanai, Samir Dutta, Glen Mola, WY Nicola Man, Anthony Zwi, C Raina MacIntyre

**Affiliations:** 1 World Health Organization, Port Moresby, Papua New Guinea; 2 School of Public Health and Community Medicine, Faculty of Medicine, University of New South Wales, Sydney, New South Wales, Australia; 3 Angau Memorial Hospital, Lae, Papua New Guinea; 4 National Department of Health, Port Moresby, Papua New Guinea; 5 Pathology Department, Port Moresby General Hospital, Port Moresby, Papua New Guinea; 6 University of Papua New Guinea, Port Moresby, Papua New Guinea

**Keywords:** Cholera, Risk factor, Sensitivity and specificity, Diagnostic accuracy, Rapid diagnostic test, Papua New Guinea

## Abstract

**Background:**

Cholera is newly emergent in Papua New Guinea but may soon become endemic. Identifying the risk factors for cholera provides evidence for targeted prevention and control measures.

**Methods:**

We conducted a hospital-based case–control study to identify cholera risk factors. Using stool culture as the standard, we evaluated a cholera point of care test in the field.

**Results:**

176 participants were recruited: 54 cases and 122 controls. Independent risk factors for cholera were: being over 20 years of age (aOR 2.5; 95%CI 1.1, 5.4), defecating in the open air (or river) (aOR 4.5; 95% CI 1.4, 14.4) and knowing someone who travelled to a cholera affected area (aOR 4.1; 95%CI 1.6, 10.7); while the availability of soap for handwashing at home was protective (aOR 0.41; 95%CI 0.19, 0.87). Those reporting access to a piped water distribution system in the home were twice as likely to report the availability of soap for handwashing. The sensitivity and specificity of the rapid test were 72% (95% CI 47–90) and 71% (95%CI 44–90%).

**Conclusions:**

Improving population access to the piped water distribution system and sanitation will likely reduce transmission by enabling enhanced hygiene and limiting the contamination of water sources. The One step V. cholerae O1/O139 Antigen Test is of limited utility for clinical decision making in a hospital setting with access to traditional laboratory methods. Settlement dwellers and mobile populations of all age groups should be targeted for interventions in Papua New Guinea.

## Background

The emergence of cholera in Papua New Guinea in July 2009 and its subsequent spread to neighboring provinces has provided significant challenges to health authorities [1]. Understanding the risk factors for cholera is important to enable the implementation of targeted prevention and control measures and to provide compelling specific evidence to government infrastructure decision makers. As of May 2011, Papua New Guinea has recorded over 15,000 cases of cholera at a case-fatality ratio of 3.2% [1]. Many of these cases presented to the longest running treatment centre in the country at Angau Memorial Hospital. Despite awareness campaigns and small scale water and sanitation interventions to reduce cholera transmission in the catchment area of this provincial referral hospital, transmission has continued since the beginning of the outbreak.

Contaminated water and food are the most common routes of cholera transmission during outbreaks [2]. Further risk factors include rainfall, seasonality, poor sanitation, travel, conflict, population displacement, health facilities with poor infection control and living near contaminated water sources. Funerals are often implicated in cholera transmission [3-5]. Cholera risk factors in the rural community setting in Papua New Guinea have been previously reported [6]; however there is no information on cholera risk factors in the urban setting, where cholera transmission has been most sustained, especially in the informal settlements.

Globally, the use of cholera rapid antigen point-of-care (POC) testing is widespread, as they can be a useful adjunct to outbreak surveillance when combined with traditional laboratory methods [7], and may enable faster outbreak identification and characterization. Earlier intervention during cholera outbreaks in newly affected areas may lead to a decrease in transmission and case fatality ratio [7], as many deaths typically occur early, when supplies, awareness, clinical skills and human resource numbers may require strengthening. While cholera POC tests have been evaluated in controlled research environments, a key question remains as to how well these tests performs directly in the hands of primary healthcare providers at a patient's bedside in crisis situations [8]. Traditional laboratory methods were nationally recommended during the Papua New Guinea cholera outbreak, however one POC test became widely available and was used for a variety of clinical and public health purposes. Definitive guidance on the utility of this POC test, which has not previously been field evaluated, and other POC tests will enable better decision making.

We report on the risk factors for cholera identified through a prospective hospital based case–control study conducted to understand disease transmission and galvanise public health action. We also discuss the field evaluation of a POC test to understand its utility in the management of a cholera epidemic.

## Methods

### Setting

Cholera was confirmed among the increasing number of cases of acute watery diarrhoea presenting to Angau Memorial Hospital from 22 August 2009. The hospital is located in Lae, the second largest city in Papua New Guinea: Lae is surrounded by an extensive network of peri-urban informal settlement areas with limited access to water and sanitation. The hospital catchment area includes the 200,000 inhabitants of Lae city and surrounding villages. Approval for this study was obtained from the Angau Memorial Hospital research committee. Written informed consent was obtained from all participants or their parent or guardians.

### Case–control study

A prospective hospital-based case–control study was conducted from 13 April to 25 June 2010. To enable the evaluation of all possible risk factors, including age, we elected to conduct an un-matched study. Suspected cases were consecutively recruited from the cholera treatment centre established on the hospital grounds. The national case definition for suspected cholera cases was used (based on the WHO definition) and was defined as a patient of any age with acute watery diarrhoea presenting to the cholera treatment centre. A confirmed case was defined as a suspected case with *V*. *cholerae* isolated from the stool. For each case, we aimed to recruit three controls and to conduct interviews within 48 hours of the interviewed case. We excluded any controls currently experiencing an episode of acute watery diarrhoea. In addition, we followed established methods and excluded all controls presenting to the hospital with illnesses other than malaria or pneumonia [9], as documented in the hospital register. Demographic, clinical, laboratory, environmental and behavioural data were collected by a registered nurse using a standardized questionnaire. All potential risk factors investigated have been documented and reported. Potential risk factors for investigation identified for inclusion in the questionnaire were from both the literature and ongoing field assessments conducted during the previous months of cholera outbreak response in this province.

### Data management

Data were entered into Microsoft Excel for cleaning and coding. Univariate analyses were performed to estimate the odds ratio (OR) and binary logistic regression to estimate the adjusted odds ratios (aOR) as well as the confidence intervals (95% CI) of variables associated with cholera using STATA version 10 (Stata Corp., College Station, TX, USA). Overall significance of the model was assessed by the likelihood ratio χ2 test and goodness-of-fit was assessed using the Hosmer-Lemeshow test [10].

### Laboratory

Stool culture was the diagnostic methodology for our case control study. However, due to the availability and usage of cholera POC tests by the cholera treatment centre staff, both the POC tests and stool culture were in use at the cholera treatment centre when the study began. Diagnostic samples for both methods were collected shortly after a case’s presentation to the facility. Testing was not performed if administration of antimicrobial therapy had already commenced. Rectal swabs were collected in Cary Blair transport media [11] and initially sent to Pathology at Port Moresby General Hospital, and in the latter part of the investigation to Angau Memorial Hospital laboratory where serological and biochemical confirmatory testing was performed. Rectal swabs were plated directly onto thiosulfate citrate bile salt sucrose (TCBS) agar (Oxoid Ltd, England). The swabs were also inoculated into alkaline peptone water (pH 6.8) for enrichment of vibrios and incubated for 6 – 8 hours then plated onto TCBS. These were incubated at 37°C for 18 to 24 hours. Typical yellow colonies of *V*. *cholerae* were biochemically tested and confirmed by agglutination with polyvalent O1 and monovalent Ogawa and Inaba antisera (Remel Europe Ltd. U.K.). Non-agglutinating strains were tested with antiserum to *V*. *cholerae* O139 strain. Antimicrobial susceptibility testing was performed according to the Kirby-Bauer method using standard discs (Oxoid Ltd. England). Laboratorians performing stool culture were unaware of the results of cholera POC tests.

### Diagnostic test evaluation

POC test kits (One step *V*. *cholerae* O1/O139 Antigen Test, Standard Diagnostics, Inc., Kyonggi-do, Korea) were used to diagnose cholera on presentation at the treatment facility. A medical doctor conducted a short training session for the nursing officer that performed the POC diagnostic tests using the immunochromatographic assay. This was her first experience using POC tests. The interval from performing POC testing to stool culture was not measured. However, due to the long duration of viability of cholera in Cary Blair transport media and the relatively short interval from sample collection to testing, we would not expect this to impact on our estimates. Using stool culture as the gold standard, the performance of the POC test was compared to generate summary statistics for diagnostic tests using the diagti module [12] in STATA version 10 (Stata Corp., College Station, TX, USA).

## Results

### Case–control study

176 participants were consecutively recruited into the study: 54 suspected cholera cases (45% culture confirmed) and 122 controls. There were no significant differences between the two groups with respect to their gender, proportion under 5 year of age, time to travel to the hospital, educational attainment and employment status (Table 1).

**Table 1:** **Demographic characteristics of cholera outbreak investigation participants**, **Papua New Guinea**, **2010**

**Demographic characteristics**		**Cases**		**Controls**	
		**n**	**(%)**	**n**	**(%)**
**Gender**	**Female**	26	(48)	67	(55)
**Age group**	**Under 5**	14	(26)	48	(39)
**Time to hospital**	**0**-**29 minutes**	39	(72)	70	(57)
	**30**-**59 minutes**	8	(15)	28	(23)
	**60 minutes** +	3	(6)	14	(11)
**Education**	**No formal**	8	(21)	16	(24)
	**Start primary**	9	(23)	11	(16)
	**Other**	22	(56)	40	(60)
**Employed**	**Yes**	13	(35)	22	(40)

Univariate analysis of recent exposures identified several possible risk factors for cholera. Cases were more likely to drink water from the river (OR 2.5; 95%CI 1.2, 5.2), and more likely to defecate in the river or field (OR 7.4; 95%CI 2.3, 27.9). Cases were also much more likely to live in an informal settlement (OR 3.2; 95%CI 1.4, 7.9) and know someone who travelled to an affected area (Table 2). Those with access to a piped water distribution system in the home were 2.5 times more likely to report soap for hand washing in the home.

**Table 2:** Cholera risk factors, Angau General Hospital, Papua New Guinea, 2010

**Risk Factor**	**Cases n=54**		**Controls n=122**		**Odds ratio**	**Confidence interval**	**p value**	**Adjusted odds ratio**	**Confidence interval**	**p value**
	**n**	**(%)**	**n**	**(%)**	(**OR**)	**(95%CI)**		**(aOR)**	**(95%CI)**	
**Over 20 years of age**	17	(32)	65	(53)	2.4	(1.2, 5.2)	0.007	2.7	(1.2, 5.8)	0.012
**Resides in an informal settlement**	44	(82)	70	(57)	3.2	(1.4, 7.9)	0.002	NA	NA	NA
**River as drinking water source**	25	(46)	31	(25)	2.5	(1.2, 5.2)	0.006	NA	NA	NA
**Defecates in open air** (**or river**)	13	(24)	5	(4)	7.4	(2.3, 27.9)	0.0001	4.6	(1.4, 14.9)	0.011
**Has soap for hand washing at home**	18	(33)	66	(54)	0.42	(0.20 0.87)	0.01	0.41	(0.19, 0.87)	0.021
**Chews betel nut**	30	(75)	43	(63)	1.74	(0.68, 4.67)	0.2	NA	NA	NA
**Washes hands before eating**	17	(32)	54	(49)	0.48	(0.23, 1.01)	0.035	NA	NA	NA
**Knows case of cholera**	16	(30)	11	(9)	4.3	(1.7, 11.0)	0.0005	2.4	(0.9, 6.2)	0.075
**Attended funeral**	5	(9)	12	(10)	0.93	(0.24, 3.02)	0.9	NA	NA	NA
**Knows someone who travelled to cholera area**	47	(87)	68	(56)	5.3	(2.2, 15.0)	0.0001	4.5	(1.8, 11.7)	0.002
**Shares housing with diarrhoea case**	11	(20)	6	(5)	5.0	(1.6, 17.2)	0.001	NA	NA	NA

The binary logistic regression model for suspected cholera (χ^2^=37.6, df=5, p<0.001 for overall significance of model) is shown in Table 2. The chosen model is not significantly better than the full model fitting all variables that were significant in the bivariate model in Table 2 (χ^2^=4.8, df=5, p=0.44). The Hosmer-Lemeshow test also indicated a good fit of the data to the model (χ^2^=3.2, df=8, p=0.92). The model identified being over 20 years of age (aOR 2.5; 95%CI 1.1, 5.4), defecating in the open air (or river) (aOR 4.5; 95% CI 1.4, 14.4) and knowing someone who travelled to a cholera affected area (aOR 4.1; 95%CI 1.6, 10.7) as being independent risk factors for cholera and having soap for hand washing at home (aOR 0.41; 95%CI 0.19, 0.87) as a protective factor (Table 2).

### Diagnostic test evaluation

Of the 54 suspected cases, diagnostic testing was performed on 53 (98%) stool samples. Of the 51 that were tested by stool culture, 23 (45%) cases were *V*. *cholerae* O1 El Tor Ogawa positive. Of the 36 tested by POC rapid antigen test, 18 (50%) were positive. The 35 samples that were tested by both stool culture and POC test were used to evaluate the POC test. The sensitivity of the POC test kits was 72% (95% CI 47–90) compared with stool culture, while the specificity was 71% (95%CI 44–90%). Antibiotic sensitivity profile demonstrated sensitivity to ampicillin, co-trimoxazole, choloramphenicol, tetracycline, naladixic acid, azithromycin, erythromycin, norfloxacin and ciprofloxacin.

## Discussion

Improving water, sanitation and other infrastructure has been associated with a 39% decline in waterborne disease in informal urban settlements in Africa [13]. The water and sanitation related Millennium Development Goals will not be achieved in Papua New Guinea [14], with the growing urban populations that dwell in critical water catchment areas a cause for concern [15]. While access to a piped water distribution system was protective on univariate analysis, it did not remain significant on multivariate analysis. Importantly, those with access to a piped water distribution system were twice as likely to report soap for handwashing in the home and cases were more than three times as likely to reside in informal settlements.

Handwashing with soap and water protects against cholera [16], and may become increasingly important during outbreaks as cholera contamination becomes widespread and disease dissemination is through multiple vehicles [17]. In our study, reporting the presence of soap for handwashing in the home was associated with protection against cholera (aOR 0.41; 95%CI 0.19, 0.87). Given that sharing a house with an infectious cholera case is a risk factor [18], improving access to soap for handwashing in the home for cholera cases that are often still infectious on discharge from treatment facilities will likely limit further transmission.

The lack of access to latrines has been identified as a risk factor for cholera in informal settlement areas in previous studies [16] and in our investigation, where cases of cholera were more likely to defecate in the open air or river than controls (aOR 4.5; 95% CI 1.4, 14.4). Months into the outbreak, half the cases were still drinking untreated river water and almost a quarter were defecating in the same rivers or open fields. While the evidence of impact of improved sanitation is weakest of the three areas of intervention to reduce transmission of diarrheal disease, it may still be effective [19] and appears highly warranted in this setting.

Evaluations of cholera POC tests conducted in controlled research environments with high levels of training have estimated sensitivity to range from 83 to 97%, and specificity from 71 to 95% [7,8,20,21]. Field evaluations have estimated the sensitivity and specificity of POC tests at 80% and 77% early in their use, improving over time [7]. Our evaluation, the first of this POC test, was conducted under field conditions during an outbreak where the user was not highly trained or experienced with POC tests. The specificity was within the range seen with other POC tests (71%; 95% CI 44–90); however the sensitivity was low (72%; 95% CI 47–90), indicating they should not be used for case management decisions (treatment or triage). Training in the use and interpretation of POC tests remains important [20], as well as the need to perform such tests alongside traditional laboratory methods in the hospital setting.

High population mobility in Papua New Guinea has been shown to impact on the risk of infectious disease transmission [22,23]. Cholera is frequently reported in association with international travel [24], however the high internal population mobility in Papua New Guinea, in the context of limited access to water and sanitation, presents an important threat to previously unaffected areas. Knowing someone who has travelled to a cholera affected area was a risk factor for cholera in our study (aOR 4.1; 95%CI 1.6, 10.7).

This study has several limitations. Similar to previous studies [25], we isolated cholera from only half the suspected cholera cases by stool culture. This may in part be explained by studies which have shown that isolation failure from suspected cholera stools by stool culture during acute diarrhoea outbreaks may be explained by the inactivation of *V*. *cholerae* by in vivo vibriolytic action of the phage and/or nonculturability induced as a host response [26]. Similar to previous studies that have relied on a high positive predictive value of the clinical case definition during cholera outbreaks [4,5,17,27-30], we did not exclude cases on microbiological confirmation of non-cholera aetiologies. The estimates of sensitivity and specificity of the POC test had wide confidence intervals due to a small sample size, however interpretation remained feasible. The sensitivity of the cholera POC test in our study may be a reflection of several factors, including the sample size and the limited training in the use and interpretation of the cholera POC tests. However the results provide insight into their utility in a typical field setting where training and experience may be limited prior to POC test usage. Three controls were not recruited for each case, however this only slightly reduced the power of the study, highlighted by the retrospective power calculations which indicate that power for a bivariate analysis was only reduced from 91% to 89% for an alpha of 5%, exposure of 50% in cases (which approximates the proportion of cases having river as a drinking water source) and minimum odds ratio of 3 when recruitment ratio was reduced from 3 (the targeted ratio) to 2.25 (the current ratio). This reduction in recruitment ratio also did not impede the identification of statistically significant risk factors in the multivariate model.

The slow progress improving water and sanitation infrastructure, in the context of the licensing and marketing of new low-cost oral cholera vaccines and the current vaccine trials in Haiti [31], may stimulate further analysis of the utility of vaccination as a component of future prevention and control in Papua New Guinea. While reactive vaccination has demonstrated its effectiveness in outbreaks in stable populations [32], many of the issues highlighted for consideration prior to implementing vaccination campaigns in Haiti [33] would be similar to those for Papua New Guinea. In our study, which was conducted in a newly affected area, cases were all ages, and were more likely than controls to be over 20 years of age (aOR 2.5; 95%CI 1.1, 5.4).

## Conclusions

Improving population access to the piped water distribution system and sanitation will likely reduce transmission by enabling enhanced hygiene and limiting the contamination of water sources. The One step *V*. *cholerae* O1/O139 Antigen Test is of limited utility for clinical decision making in a hospital setting with access to traditional laboratory methods. Settlement dwellers and mobile populations of all age groups should be targeted for interventions in Papua New Guinea.

## Abbreviations

aOR: Adjusted odds ratio; CI: Confidence interval; OR: Odds ratio; POC: Point of care; TCBS: Thiosulfate citrate bile salt sucrose; *V*. *cholerae*: *Vibrio cholerae*.

## Competing interests

The authors declare that they have no competing interests.

## Authors’ contributions

AR made substantial contributions to conception and design, analysis and interpretation of data and was involved in drafting the manuscript. BA made substantial contributions to acquisition and interpretation of data and has been involved in drafting the manuscript. LK made substantial contributions to acquisition of data. FM has been involved in drafting the manuscript. BR made substantial contributions to conception and has been involved in drafting the manuscript. EP made substantial contributions to conception and has been involved in drafting the manuscript. SD made substantial contributions to acquisition of data. GM was involved in drafting the manuscript and revising it critically for important intellectual content. WYNM made substantial contributions the analysis and interpretation of data and has been involved in critically revising the manuscript for important intellectual content. AZ was involved in drafting the manuscript and revising it critically for important intellectual content. CRM made substantial contributions to analysis and interpretation of data and has been involved in revising it critically for important intellectual content. All authors read and approved the final manuscript.

## Pre-publication history

The pre-publication history for this paper can be accessed here:

http://www.biomedcentral.com/1471-2334/12/287/prepub
